# Ictal Mammalian Dive Response: A Likely Cause of Sudden Unexpected Death in Epilepsy

**DOI:** 10.3389/fneur.2018.00677

**Published:** 2018-08-17

**Authors:** Jose L. Vega

**Affiliations:** ^1^Department of Neurosciences and Stroke, Novant Health, Forsyth Medical Center, Winston-Salem, NC, United States; ^2^TeleNeurologia SAS, Medellin, Colombia

**Keywords:** sudden unexpected death in epilepsy, SUDEP, mammalian dive response, MDR, pulmonary edema, diving bradycardia, apnea, demargination

## Abstract

Even though sudden unexpected death in epilepsy (SUDEP) takes the lives of thousands of otherwise healthy epilepsy patients every year, the physiopathology associated with this condition remains unexplained. This article explores important parallels, which exist between the clinical observations and pathological responses associated with SUDEP, and the pathological responses that can develop when a set of autonomic reflexes known as the mammalian dive response (MDR) is deployed. Mostly unknown to physicians, this evolutionarily conserved physiological response to prolonged apnea economizes oxygen for preferential use by the brain. However, the drastic cardiovascular adjustments required for its execution, which include severe bradycardia and the sequestration of a significant portion of the total blood volume inside the cardiopulmonary vasculature, can result in many of the same pathological responses associated with SUDEP. Thus, this article advances the hypothesis that prolonged apneic generalized tonic clonic seizures induce augmented forms of the MDR, which, in the most severe cases, cause SUDEP.

## Introduction

Increasingly, neurologists are coming to terms with the reality that epilepsy patients die suddenly 24–28 times more frequently than the general population (1, 2). Victims are often found dead, in bed. Seizures are occasionally witnessed prior to death, but often only a hint of one is discovered (e.g., a tongue bite) (3). Witness accounts indicate that sudden unexpected death in epilepsy (SUDEP), as this enigmatic phenomenon is known, is associated with generalized tonic-clonic seizures (GTCS) (4–7), hypoventilation, peri-ictal apnea (5, 8–10), bradycardia, ictal asystole (10), and postictal generalized electroencephalographic suppression (PGES) (11). From an epidemiological standpoint, SUDEP affects patients who are young and otherwise healthy (12–14), as well as patients who suffer from comorbid organic psychiatric disease (14).

A well-known study of SUDEP cases videotaped inside epilepsy monitoring units (EMU) from around the world demonstrated a consistent pattern that involves GTCS, ictal hyperventilation, cardiorespiratory dysfunction, and terminal apnea followed by bradycardia and asystole (10). Autopsy reports have shown pulmonary edema so consistently, (15–19) that it is considered a pathological hallmark of this condition (5, 10). Pulmonary hemorrhages, cerebral edema and/or cerebral petechial hemorrhages are also found at autopsy, although less frequently (15, 17–19). Heart and lung weights are generally increased, in association with a normal cardiac structure (12, 15, 18), suggesting that SUDEP results from an acute process unrelated to chronic cardiac disease.

As SUDEP is relatively rare and difficult to investigate (20), it is not entirely surprising that these clinical observations and pathological findings remain unexplained. Current theories include a convergence of postictal coma, airway obstruction and hypoventilation (9), central and obstructive apnea (5), susceptibility to sudden death due to the coexistence of genetic cardiac arrhythmogenicity and epilepsy (21), postictal neurovegetative breakdown (10), and imbalances in sympathetic and parasympathetic control over cardiorespiratory function (22). While these theories offer a general view of the suspected physiopathology of SUDEP, a mechanistic hypothesis is lacking.

This article provides a brief description of the mammalian dive response (MDR), a set of autonomic reflexes triggered simultaneously during sustained apnea in order to protect the brain from hypoxia. It also presents remarkable parallels which exist between the physiopathology of this response, and that of SUDEP, and hypothesizes that prolonged apneic seizures induce augmented, ictal, forms of the MDR, which can culminate in sudden death.

## What is the MDR?

Humans share with marine and other terrestrial mammals a multipronged, autonomic response to submersion known as the MDR (23, 24). Its discovery dates back to 1786 when Edmund Goodwyn [1756–1829], an inquisitive medical student at the University of Edinburgh, subjected a toad to conditions of forced water immersion while investigating the physiological correlates of death by drowning (25, 26). He observed that quickly after immersion, the toad's heart rate decelerated gradually, until it ceased. But soon after its removal from the water, the toad took a deep breath, its heart resumed beating, and it walked about “without any expressions of uneasiness” (25). Goodwyn, however, did not realize the importance of this observation, which remained in relative obscurity for several decades until it became part of the theoretical background that led Paul Bert [1833–1886] to find a similar phenomenon in ducks (26, 27). Later, Charles Richet [1850–1935] demonstrated that Bert's observation represented an oxygen-conserving reflex which is triggered when water makes contact with its nostrils and beak (28). In addition, by blocking the reflex with atropine, he was able to attribute its efferent arm to the decelerating action of the vagus nerve on the heart (29). Decades later, Andersen and colleagues performed selective denervation experiments which led them to conclude that the afferent arm of the reflex was mediated by the ophthalmic branch of the trigeminal nerve (30). Today, over 230 years after its first description, we know that this primitive, oxygen-conserving reflex, which has come to be known as “diving bradycardia” (31, 32), is only one aspect of the autonomic nervous system's response to submersion, –the MDR– which also involves apnea, peripheral vasoconstriction, and splenic contractions (24, 31).

The apnea of the MDR was first shown in anesthetized and decorticated ducks subjected to forced water immersion (33), but a similar reflex was subsequently found in several marine and terrestrial species (34–38). This involuntary apnea, which occurs simultaneously with diving bradycardia, is also associated with a profound vasoconstriction that prevents blood from circulating through peripheral tissues, thus conserving most of the available oxygen for preferential use by the brain and heart (34, 35, 39–41). Seals, for instance, experience over 90% reduction in peripheral circulation in association with a nearly 400% increase in cardiopulmonary blood flow during submersion (41). While cardiac output is reduced under these circumstances, cerebral blood flow is preserved (41) or increased (42). A fourth reflex, also triggered simultaneously, was discovered when seals were noted to exhibit hematocrit increases of more than 60% during some dives (43, 44). This effect was later found to stem from a release of resident red blood cells (RBCs) from the spleen, due to contractions promoted by systemic catecholamines (45, 46). The resulting boost in hemoglobin from these contractions helps to maximize brain oxygenation during apnea (47). Thus, the MDR is composed of four oxygen-conserving reflexes –apnea, diving bradycardia, peripheral vasoconstriction, and splenic contractions, –which act simultaneously during submersion to protect the brain from hypoxia (23, 24, 31, 48).

## The human MDR

After decades of animal research on the subject, three legendary physiologists, Irving, Sholander and Grinnell, finally reported “a good human diver” who exhibited significant diving bradycardia while swimming (49). Several years later it was shown that humans can elicit diving bradycardia by combining facial immersion with voluntary apnea (50–52). This effect occurs simultaneously with a marked peripheral vasoconstriction (53, 54), which shunts a significant portion of the total blood volume into the cardiopulmonary vasculature (55–58). This latter component of the human MDR is often referred to as the “blood shift” (55, 56, 59). However, unlike aquatic mammals, whose blood pressure is unchanged by the MDR (60), human blood pressure increases drastically by this response, reaching values as high as 280/200 (61, 62). Such an altered hemodynamic state increases cerebral blood flow (62–65) and impairs cerebral autoregulation (66), despite a concomitant reduction of cardiac output (61, 67). The human MDR also involves splenic contractions that result in transient but modest elevations in circulating RBCs (45, 68, 69). A bystander effect of these contractions, without any apparent physiological benefit to the oxygen-conserving purposes of the MDR, is the release of large numbers of splenic white blood cells (WBC) into the bloodstream (68). More recent work has shown that humans can trigger the MDR through prolonged voluntary apnea, independently of facial immersion (62, 63, 65, 69), and this effect is augmented if apnea is performed with exercise (51, 54, 65, 70). Thus, humans, like all other vertebrate species tested to date, exhibit a complex oxygen-conserving response to prolonged apnea, the MDR, which is characterized by bradycardia, vasoconstriction, hypertension, increased cardiopulmonary and cerebral blood flow, and splenic contractions (48).

## Physiopathology of the human MDR

Even though the MDR represents a beneficial set of reflexes to conserve oxygen and maximize the chances of survival during apneic conditions (23, 24), its deployment poses a major challenge to human physiology. For instance, the overwhelming parasympathetic discharge that causes diving bradycardia can result in heart rates as low as eight beats per minute, and is invariably accompanied by electrocardiographic abnormalities, including pointed T waves, ectopic beats, abnormal P waves, atrioventricular nodal and idioventricular rhythms, AV block, sinus-arrest followed by nodal or ventricular escape, and ectopic ventricular beats (31, 71–73). Combined with a simultaneous, and equally overwhelming sympathetic response, which induces severe peripheral vasoconstriction (51, 54), and hypertension (61, 62, 64) the MDR generates an autonomic conflict that can lead to dangerous conduction abnormalities (74). This has led some to hypothesize that the diving bradycardia of the MDR is to blame for some cases of sudden cardiac death (74, 75), and sudden infant death syndrome (76). Triggered while swimming, diving bradycardia is also thought to induce fatal cardiac arrhythmias in otherwise healthy individuals who harbor subclinical pro-arrhythmogenic conditions (77–80). For instance, an eye-opening study that tested for cardiac channelopathies in drowning victims, found that nearly 30% of them hosted mutations that would have made them susceptible to developing either long QT syndrome (LQTS) or catecholaminergic polymorphic ventricular tachycardia while swimming (79). These data are in sharp contrast with, for instance, the 0.05% estimated incidence of congenital LQTS in the general population (81), and further implicate diving bradycardia in the unmasking of potentially lethal arrhythmias (74, 75, 77–80, 82, 83).

The blood shift is yet another aspect of the MDR thought to induce pathology in humans, due to the abrupt shunting of excess blood into the intrapulmonary vasculature (55–58). Combined with the strong vasoconstriction characteristic of the MDR (53, 54), the blood shift (55, 56, 59) can result in elevated pulmonary capillary transmural pressure, and in the subsequent extravasation of fluid, or blood into the interstitium or into the alveolar space (84) (i.e., pulmonary edema, and pulmonary hemorrhage). This form of pulmonary pathology is often seen in breath-hold divers who induce the MDR by diving to significant depths under apneic conditions (85–87). In this population pulmonary edema becomes clinically manifest as shortness of breath, chest tightness, and the production of a pink, frothy sputum, or frank blood, upon surfacing from a dive (85–87). Rales or crackles, abnormally low arterial oxygen saturation, and reduced pulmonary performance are revealed by physical examination, pulse oximetry and spirometry, respectively (85, 86, 88). A strikingly similar form of pulmonary edema has also been reported in swimmers (89, 90). Thus, two of the main components of the human MDR, diving bradycardia and the blood shift, have been implicated in the development of cardiac arrhythmias, cardiac arrest, pulmonary edema, and pulmonary hemorrhage.

## Hypothesis: a significant fraction of SUDEP victims succumb to an unfettered ictal form of the MDR

As discussed above, a major effect of the MDR is the sequestration of blood inside the cardiopulmonary circulation (23, 24, 35, 55, 56). This is especially important during apneic exercise, when unrestricted blood flow to active muscles could result in a rapid depletion of the limited supply of oxygen available to the brain. It is therefore appropriate that apnea associated with exercise induces an augmented form of the MDR by comparison to that induced by apnea at rest (51, 54, 65, 70). Thus, it is hypothesized that apneic GTCS (91, 92), which by definition exhibit vigorous muscle activity, pose physiological challenges that result in augmented, ictal forms of the MDR. It is further hypothesized that unlike a MDR deployed in a conscious subject, which could be tamed by higher cortical function (75, 93), an ictal MDR deployed during GTCS, or during a period of electrographic suppression (10, 11, 94), has a greater potential to become pernicious, and induce one or more of the common pathological findings of SUDEP, namely pulmonary edema, pulmonary hemorrhage, cerebral edema, cerebral petechial hemorrhage, or cardiac arrest (see Table 1). Specifically, over-distension of pulmonary blood vessels brought on by an augmented blood shift (56, 57, 59), in combination with simultaneous and pronounced vasoconstriction (53, 54, 63, 64), can increase pulmonary capillary transmural pressure to the point of precipitating pulmonary edema and pulmonary hemorrhage (15, 17–19, 84). The magnitude of this effect is likely to correlate with the degree of MDR augmentation produced by specific seizure characteristics. For instance, a non-convulsive seizure that features a brief period of sustained apnea starting at the peak of the lung's inspiratory reserve volume is likely to induce a lesser degree of pulmonary edema or hemorrhage than a convulsive seizure that features a prolonged period of sustained apnea starting at the nadir of the tidal volume. A more severe form of pulmonary edema could occur when a prolonged ictal cry (i.e., a sustained expiratory laryngeal vocalization), precedes the onset of apnea, as the MDR is augmented if triggered at the end of expiration (106, 107). Perhaps the worst pulmonary pathology is to be expected when a prolonged apneic GTCS preceded by an ictal cry is followed by a forceful inhalation against a blocked airway (e.g., from positional causes resulting from the convulsion (9) or from laryngospasm (108)), as this would decrease intra-alveolar pressure further, resulting in the extravasation of fluid and blood into the alveoli (i.e., negative pressure pulmonary edema) (84, 109).

**Table 1:** Hypothesized pathological effects of ictal MDRs.

**Documented physiologic MDR**		**Hypothesized ictal MDR**	
**Physiologic MDR**	**Pathologic MDR**	**Non-lethal GTCS**	**SUDEP**
**APNEA** James and De Burgh Daly (36) Huxley (33)	**Prolonged voluntary apnea** **Loss of consciousness** Lindholm and Lundgren (87)	**Transient involuntary apnea** Goldenholz et al. (92) Nashef et al. (91)	**Terminal apnea** Ryvlin et al. (10) Tao et al. (9) Langan et al. (5) So et al. (8)
**Diving bradycardia** Irving et al. (49) Bert (27) Goodwyn, (96)	**Transient arrhythmias** Wierzba et al. (73) Elsner and Gooden (31) Olsen et al. (72) Wyss (71)	**Transient bradyarrhythmias** Hampel et al. (95) Fava et al. (97) So and Sperling (98) Reeves et al. (99) Nashef et al. (91)	**Terminal bradycardia** **Cardiac arrest** Ryvlin et al. (10)
**Blood shift** Schagatay (59) Andersson et al. (54) Arborelius et al. (57) Arborelius et al. (58) Schaefer et al. (55) Craig (56)	**Pulmonary edema** **Pulmonary hemorrhage** Lindholm and Lundgren (87) Lindholm et al. (85) Liner and Andersson (86)	**Neurogenic pulmonary edema** Kennedy et al. (100) **Transient lactic acidosis** Lipka and Bulow (101) Orringer et al. (102) **Transiently increased ICP** Solheim et al. (103)	**Pulmonary edema** Nascimento et al. (19) Esen Melez et al. (18) Antoniuk et al. (17) Kloster and Engelskjon (16) Terrence et al. (15) **Pulmonary hemorrhage** **Cerebral petechial hemorrhage** **Cerebral edema** Esen Melez et al. (18) Nascimento et al. (19) Antoniuk et al. (17) Terrence et al. (15)
**Splenic contraction** Schagatay et al. (69) Bakovic et al. (68)	NRPF	**Transient Leukocytosis** Aydogan et al. (104) Shah et al. (105)	NRPF

*The four reflexes that make up the MDR are shown under the “Physiologic MDR” column. Known physiopathological effects associated with each of these four reflexes in humans are shown under the “Pathologic MDR” column. The right half of the table shows reported clinical and pathological observations associated with non-lethal GTCS, and SUDEP, in relation to their hypothetically causative ictal MDR reflexes. Literature cited is merely representative. NRPF, No related publications found*.

Aside from providing an explanation for the pulmonary pathology seen in SUDEP, the expanded intrathoracic blood volume generated by the blood shift of the MDR also accounts for the increased lung weights frequently observed in SUDEP autopsies (15, 17). However, it does not account for the increased heart weight, as blood is typically emptied from the atria and ventricles before the heart is weighed at autopsy. Similarly, this hypothesis does not intuitively explain the presence of focal myocardial fibrosis previously seen in some SUDEP autopsies (19).

If the hypertension that occurs during the human MDR (61) is increased further during an apneic GTCS, it could induce cerebral edema and/or cerebral petechial hemorrhage (17, 18) by increasing cerebral perfusion (62–65) to a critical level that disrupts cerebral vascular autoregulation (66). This notion is consistent with previous demonstrations of increased intracranial pressure (103), and increased sympathetic overactivity during seizures (110, 111), including a recent report of a probable SUDEP victim in whom excess sympathetic activity coexisted with terminal apnea for several minutes preceding death (112). Theoretically, a MDR preceded by intense hyperventilation (i.e., prolonged apnea preceded by hyperventilation), such as that seen in the majority of SUDEP cases reported by Ryvlin and colleagues (10), could further exacerbate this process by lowering arterial CO_2_ levels, and thus inducing cerebral vasoconstriction before ictal MDR deployment.

While, theoretically, pulmonary edema and/or pulmonary hemorrhage, in combination with cerebral edema or cerebral hemorrhage could lead to SUDEP, the autonomic conflict generated by the simultaneous parasympathetic and sympathetic influences on the heart can also lead to SUDEP by inducing ventricular arrhythmias (74). This pro-arrhythmogenic state (95, 97–99, 113) could provoke sudden death in some epilepsy patients –not always accounted as SUDEP—who consume substances that prolong the QT interval, including recreational ones such as alcohol and amphetamines, and therapeutic ones such as citalopram, and quetiapine (82, 83, 114). Furthermore, the MDR can act as an “effect amplifier” (115), responsible for SUDEP in patients who harbor clinically silent pro-arrhythmogenic gene mutations (116). Finally, the high incidence of SUDEP among the young (12–14) is congruent with reports that the MDR is strongest in younger age groups (73, 117, 118).

If, as suggested by the present hypothesis, ictal MDRs are normally triggered by prolonged apneic seizures, then all such seizures should demonstrate a predetermined potential to induce SUDEP, or SUDEP-related pathology, depending on the magnitude of their associated ictal MDR. This notion is supported by several clinical observations familiar to most neurologists (see Table 1). For instance, GTCS induce “neurogenic” pulmonary edema in some patients, through an effect that is directly related to seizure duration (100), suggesting that prolonged, intense, apneic convulsions might be more likely to induce pathogenic forms of the ictal MDR than short, mild ones. If this is true, the possibility arises that “spontaneous” neurogenic pulmonary edema is not actually spontaneous, but instead, is triggered by *unwitnessed*, prolonged apneic GTCS. After all, this form of pulmonary edema is often reported in patients who are found unconscious or with different degrees of altered mentation (i.e., possibly postictal) before being transported to the hospital, and who harbor epileptogenic pathologies, such as brain tumors (119), hydrocephalus (120), subdural hematomas (121), strokes (122), and subarachnoid hemorrhages (123, 124). Similarly, GTCS often induce a short-lived lactic acidosis (101, 102), which is comparable to that displayed by air-breathing aquatic mammals (34), and humans (61), surfacing from long dives. This lactic acidosis is precipitated by the blood shift, which as stated previously, restricts blood flow to active muscles and forces them to undergo anaerobic metabolism (34, 61). Consequently, it is possible that the magnitude of an ictal blood shift could be responsible for at least some of the characteristics of the transient lactic acidosis seen after GTCS (e.g., peak lactic acid level, duration, etc.). Finally, GTCS occasionally induce a transient leukocytosis (104, 105), which is thought to reflect a release of granulocytes from perivenular locations in response to systemic catecholamines (125). The present hypothesis suggests that the splenic contractions triggered during an ictal MDR (68) could play a role in generating this leukocytosis.

Because frank cessation of breathing is required to trigger the various autonomic components of the MDR, the present hypothesis cannot adequately explain cases in which SUDEP occurs independently of apnea. In addition, this hypothesis fails to explain SUDEP cases characterized by sustained hypotension, as by definition the human blood shift is accompanied by severe hypertension. Unfortunately, the latter cannot be tested against published observations due to a dearth of blood pressure measurements in the SUDEP literature. Moreover, while hypertension appears to predominate over hypotension during non-lethal seizures (111), hypotension can also occur (126), preventing us from drawing any firm conclusions that could support or refute a role for the ictal MDR in SUDEP.

## How can this hypothesis be tested?

As both seizures and their postictal period can exhibit prolonged periods of apnea, (8–10, 91, 92), it should be possible to confirm whether a sequential, and causal, relationship exists between apnea and (1) bradycardia, (2) LQTS, (3) asystole or other arrhythmias, or (4) any combination of these. This can be learned from prospective observations recorded on epilepsy patients monitored for nasal airflow, chest/abdominal excursions, forehead pulse oximetry, and multichannel ECGs at the EMU. Continuous noninvasive blood pressure measurements during and after prolonged apneic GTCS can ascertain whether hypertension occurs in association with bradycardia, and whether it bears a quantifiable relationship with the blood shift, and with lactic acidosis. The latter could be documented via a combination of peripheral arterial Doppler ultrasound studies, echocardiography, chest X-rays, and blood tests performed at different times during the postictal period of prolonged apneic GTCS. If possible, extracranial and intracranial Doppler ultrasound tests can be used to determine the relationship between apnea, hypertension and cerebral blood flow during, and after, prolonged apneic GTCS. Other research questions might aim at revealing whether the magnitude of seizure-induced leukocytosis is related to the magnitude of splenic contractions. This could be accomplished by using ultrasound to correlate the absolute change in splenic size (size before vs. size after GTCS) with the corresponding peak leukocytosis after a GTCS. These studies could result in the identification of post-seizure leukocytosis as a surrogate marker for ictal MDR deployment, which in turn could be used to identify patients at risk of SUDEP. Similar research questions can be asked of post-seizure lactic acidosis. Finally, if this hypothesis were correct, it would be important to understand why some seizures induce ictal MDRs that result in SUDEP, and why others only induce leukocytosis, lactic acidosis, or mild pulmonary edema. This could be related to several factors, including total apneic time, volume of air in the lungs at the onset of apnea, arterial levels of O_2_ or CO_2_ at the onset or the end of apnea, intensity of convulsions, and electrographic characteristics, among others. Could such factors be altered during a GTCS to prevent SUDEP?

In unconscious dogs, the MDR can be aborted by artificial lung inflation (127). Could a similar intervention be used in humans to prevent SUDEP? The answer to this and other important questions related to the relationship between SUDEP and MDR physiopathology could bring us closer to understanding SUDEP, and to generating a strategic way to prevent it.

## Author contributions

The author confirms being the sole contributor of this work and approved it for publication.

### Conflict of interest statement

The author declares that the research was conducted in the absence of any commercial or financial relationships that could be construed as a potential conflict of interest.
